# HIV-1 transmission and survival according to feeding options in infants born to HIV-infected women in Yaoundé, Cameroon

**DOI:** 10.1186/s12887-018-1049-3

**Published:** 2018-02-19

**Authors:** Anne Esther Njom Nlend, Annie Carole Nga Motaze, Arsene Sandie, Joseph Fokam

**Affiliations:** 1 Pediatric Service, National Insurance Fund Welfare Hospital, Yaoundé, Cameroon; 2 Higher Institute of Medical Technology, University of Douala, Yaoundé, Cameroon; 3 Statistics and Demography, Panafrican University, Nairobi, Kenya; 4 Virology Laboratory, Chantal BIYA International Reference Centre for Research on HIV/AIDS prevention and management, Yaoundé, Cameroon; 5 Faculty of Medicine and Biomedical Sciences, University of Yaoundé 1, Yaoundé, Cameroon; 6 Chair of Virology, Faculty of Medicine and Surgery, University of Rome Tor Vergata, Rome, Italy

**Keywords:** HIV-1 vertical transmission, Survival, Feeding option, Infants, Cameroon

## Abstract

**Background:**

Evidence of 24-months survival in the frame of prevention of mother-to-child transmission (PMTCT) cascade-care is scare from routine programs in sub-Saharan African (SSA) settings. Specifically, data on infant outcomes according to feeding options remain largely unknown by month-24, thus limiting its breath for public-health recommendations toward eliminating new pediatric HIV-1 infections and improving care. We sought to evaluate HIV-1 vertical transmission and infant survival rates according to feeding options.

**Methods:**

A retrospective cohort-study conducted in Yaounde from April 2008 through December 2013 among 1086 infants born to HIV-infected women and followed-up throughout the PMTCT cascade-care until 24-months. Infants with documented feeding option during their first 3 months of life (408 on Exclusive Breastfeeding [EBF], 663 Exclusive Replacement feeding [ERF], 15 mixed feeding [MF]) and known HIV-status were enrolled. HIV-1 vertical transmission, survival and feeding options were analyzed using Kaplan Meier Survival Estimate, Cox model and Schoenfeld residuals tests, at 5% statistical significance.

**Results:**

Overall HIV-1 vertical transmission was 3.59% (39), and varied by feeding options: EBF (2.70%), ERF (3.77%), MF (20%), *p* = 0.002; without significance between EBF and ERF (*p* = 0.34). As expected, HIV-1 transmission also varied with PMTCT-interventions: 1.7% (10/566) from ART-group, 1.9% (8/411) from AZT-group, and 19.2% (21/109) from ARV-naïve group, *p* < 0.0001. Overall mortality was 2.58% (28), higher in HIV-infected (10.25%) vs. uninfected (2.29%) infants (*p* = 0.016); with a survival cumulative probability of 89.3% [79.9%–99.8%] vs. 96.4% [94.8%–97.9% respectively], *p* = 0.024. Mortality also varied by feeding option: ERF (2.41%), EBF (2.45%), MF (13.33%), *p* = 0.03; with a survival cumulative probability of 96% [94%–98%] in ERF, 96.4% [94.1%–98.8%] in EBF, and 86.67% [71.06%–100%] in MF, *p* = 0.04. Using Schoenfeld residuals test, only HIV status was a predictor of survival at 24 months (hazard ratio 0.23 [0.072–0.72], *p* = 0.01).

**Conclusion:**

Besides using ART for PMTCT-interventions, practice of MF also drives HIV-1 vertical transmission and mortality among HIV-infected children. Thus, throughout PMTCT option B+ cascade-care, continuous counseling on safer feeding options would to further eliminating new MTCT, optimizing response to care, and improving the life expectancy of these children in high-priority countries.

## Background

During the last decade, the medical advancement and breakthrough in preventing HIV pediatric infections have been widely implemented, with prevention of mother-to-child transmission of HIV (PMTCT) option B+ emerging as a key intervention in current routine practices [1–3]. As a result, PMTCT is gradually moving towards eliminating MTCT (e-MTCT) due to efforts in the universal access to antiretroviral (ARV) drugs for HIV-infected pregnant women, especially those living in the 22 high-priority countries [3]. However, despite the global commitment in e-MTCT targets, achieving HIV vertical transmission rates ≤5% in breastfeeding and ≤2% less in non-breastfeeding populations remains challenging in most sub-Saharan Africa (SSA) countries [4, 5]. Pragmatic strategies to achieving the goal of e-MTCT include, but not limited to, generalized coverage of HIV screening amongst pregnant women through provider initiated counseling and testing, and implementation of option B+ (lifelong triple ARV therapy [ART] to all HIV-infected pregnant women regardless of clinical or immunological eligibility) [3, 6–8].

Cameroon falls amongst the 21 SSA countries that are part of 22 global countries prioritized for this fight against new pediatric infections [3, 9, 10]. Bottlenecks encountered throughout the PMTCT-cascade contribute in sustaining high rates of HIV vertical transmission in general [5], in spite of promising findings in some specific sites [11]. Tackling such challenges in the era of PMTCT option B+ cascade-care requires resolving issues around retention in care and suboptimal feeding option(s), while addressing related programmatic issues that include the decentralization process [12], distance between households and the nearest health facility [13], community engagement and male-partner involvement into the PMTCT cascade-care components [14–16].

Within our catchment area, the Djoungolo Health District of Yaoundé (capital city of Cameroon), PMTCT programme started in the early 2008 with promising outcomes recorded in both breastfeeding and non-breastfeeding populations during the first half of infancy [11]. In spite of the national recommendations for monitoring HIV-exposed infants until completion of PMTCT-cascade-care, retention in care is concerning, with persistent risks of HIV-vertical transmission around 24 months of infant age (above 8% nationwide) [17]. Faced with scarcity of thorough perinatal cohorts capable of providing 24-month outcomes in children within the PMTCT-cascade care in Cameroon, it becomes relevant to generate local evidence that may help in formulating policies supporting the optimal efficacy or effectiveness of PMTCT programs in resource limited settings (RLS) like SSA. Of note, in the era of option B+, whereby considerations to ARV interventions and infant feeding practices had earlier been addressed for PMTCT [18], advanced understanding of the effects of infant feeding options, on both HIV MTCT and infant survival potentials, becomes essential for eventually improving on targets of eMTCT in high priority countries [3].

### Study objectives

We aimed to evaluate HIV-1 vertical transmission and infant survival rates according to feeding options, in a typical RLS with generalized HIV epidemiology, for possible knowledge generalizability to PMTCT high-priority countries with similar programmatic features.

## Methods

### Study design and populations

We conducted a retrospective cohort-study from April 2008 through December 2013 amongst infants born to HIV-infected women, and followed-up throughout the PMTCT cascade-care until 24-months at the Djoungolo Health District in Yaoundé-Cameroon.

### Study procedures

Patients and their respective data were retrieved from the Djoungolo PMTCT cohort for the period 2008-2013. Characteristics of this perinatal cohort were extensively described elsewhere [11].

Briefly, PMTCT program was launched in 2008 at the Djoungolo Health District, a setting within the catchment area of the Essos Hospital Centre (EHC), classified as the area HIV reference treatment center. Routinely, pregnant women diagnosed positive for HIV in primary healthcare centers of Djoungolo were referred to EHC for HIV-related clinical assessment, CD4 cell count measurement, infant feeding counseling (IFC) and ART initiation following standard-of-care [11]. Routine antenatal care (ANC) and delivery were done in their respective primary clinics. After delivery, mothers were advised to register their babies at the EHC for ART provision, postnatal follow-up and monitoring of PMTCT-related outcomes. Postnatal care consisted of HIV-1 early infant diagnosis (EID) at 6 weeks of age, clinical visits, continuous counseling on and follow-up of feeding practices, administration of Cotrimoxazole and late serological testing. Routine immunizations could be done at EHC or at another recognized health facility within the Djoungolo Health District. Routine appointments were planned at week 6, month 9, month 12, and between months 15-24. EID, performed using either real time PCR or qualitative PCR, was done at week 6 and further confirmed after month 12, considering 6 weeks of post weaning period (i.e. for breastfeeding populations).

During the study period, changes in PMTCT recommended ARVs used for pregnant women were taken into account. Before 2010, women having a CD4 count above 350 cells/mm^3^ received zidovudine (AZT) from week 28 of pregnancy till birth. As from 2010, AZT was given from week 14, while women with CD4 count below 350 cells/mm^3^ or clinically classified at the WHO stage 3 or 4 were eligible for lifelong triple ART.

### Choice of infant feeding options

Counseling for infant feeding options was part of ANC and postnatal care. Specifically, IFC was done during each ANC visit, and HIV positive women were referred to the infant feeding counselors for in-depth counseling purposes. During IFC, the two recommended exclusive options were either exclusive breastfeeding (EBF) or exclusive replacement feeding (ERF), with emphasis to avoid mixed feeding (MF) practice.

EBF was defined as breastfeeding with no added supplements except vitamins; ERF was defined as the administration of commercial formula feeding using bottles; while MF was described as a combination of both breastfeeding and formula feeding practices. Mothers opting to practice ERF were trained on the appropriate use of feeding bottles in terms of preparation, conservation and hygiene of feeding bottles and teens.

After IFC, mothers were all capable to freely make a choice of the appropriate feeding option for their babies. Additionally, supportive counseling was done during follow-up visits. EBF mothers were advised to EBF for 6 months, followed by weaning during a short period (i.e. 1 week). After 2009, the weaning period was extended to 1 month.

### Infant eligibility criteria

Infants born alive, to an HIV-infected mother, were enrolled at their first postnatal clinic attendance, specifically in the frame of EID (i.e. around week 6). The reported feeding option of mothers was recorded and those with doubtful or missing feeding information were not included. Reported feeding options were then used to classify infants as EBF, ERF or MF. During follow-up, the conclusive HIV-free result was provided upon the criteria of one negative virological + negative serological testing ≥12 months assuming 6 weeks post weaning in case of breastfeeding. A confirmed HIV infection was based on two positive PCR at any moment of the follow-up irrespective of feeding option, or a positive HIV serology after 18 months. Children with indeterminate HIV serological result were excluded from the analysis.

### Statistical analysis

Primary endpoints were HIV transmission and survival rates at 24 months of infant age. Kaplan Meier survival curves were used to estimate cumulative probabilities of survival overtime according to feeding option, and log-rank test was used to compare the curves between different feeding options. Multivariate Cox proportional hazards regression was used to estimate unadjusted and adjusted hazard ratios, adjusting for HIV serological status and birth weight. Schoenfeld residuals test which was used in assessing the proportional hazard assumption [19] and Cox-Snell residual was used to determine the goodness-of-fit. All statistical tests were performed at 5% level of significance and with 95% confidence interval. Statistical analyses were done using the package survival incorporated in R software version 3.1.3.

### Ethical statements

Ethical clearance for the study was obtained from the National Ethics Committee for Research on Human Subjects in Cameroon (2013/02/027/L/CNER/SP) and the Institutional Review Board of EHC (2014/003/CE-CHE). All women participating with their infants in the IFC project provided a written informed consent. Data were processed using unique identifiers for purposes of privacy and confidentiality.

## Results

### Characteristics of the study population

**Fig. 1:**
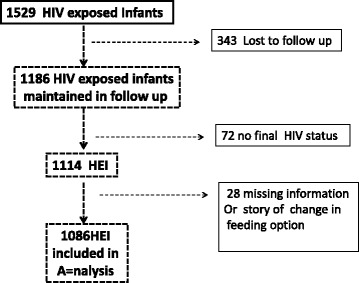
Flow chart of HIV exposed infants included in the survival analysis according to the feeding mode

A total of 1086 eligible infants born to HIV positive mothers (median of 399 [IQR: 260-540] CD4 cells/mm^3^) were enrolled (Fig. 1). Regarding maternal ARV experience for PMTCT-interventions, 566 (52.12%) received triple ART, 411 (37.85%) received AZT and 109 (10.04%) had no ARV exposure. Regarding infant feeding options during the first 3 months of life, 663 (61.05%) were on ERF, 408 (37.57%) on EBF and 15 (1.38%) on MF (Table 1).

**Table 1:** Baseline maternal and 24-months outcomes infant characteristics according to feeding mode in the HIV exposed infants of the Essos hospital center PMTCT program

	ERF *N* = 658	EBF *N* = 405	MF *N* = 14	*p*-value
Maternal characteristics				
Age mean (SD)	27.00 (5.35)	27.53 (5.18)	27.00 (4.88)	0.30
Marital status—*n*(%)				0.48
Single	153 (36.51)	96 (37.80)	6 (54.55)	
In relationship	266 (63.49)	158 (62.20)	5 (45.45)	
Parity—*n*(%)				0.67
Multiparous	278 (66.99)	176 (70.40)	8 (72.73)	
Primiparous	137 (33.01)	74 (29.60)	3 (27.27)	
CD4 cells count--mean(SD)	402.60 (230.72)	472.16 (397.74)	534.17 (107.39)	0.003
OMS Stage—*n*(%)				0.37
1	486 (96.05)	312 (94.83)	11 (100.00)	
2	18 (3.56)	11 (3.34)	0 (0.00)	
3	2 (0.40)	5 (1.52)	0 (0.00)	
4	0 (0.00)	1 (0.30)	0 (0.00)	
Occupation—*n*(%)				0.75
Student	35 (0.051)	22 (0.032)	2 (0.0029)	
Formal sector	140 (8.37)	82 (8.73)	5 (18.18)	
Informal sector	183 (33.49)	116 (32.54)	3 (45.45)	
Housewife	60 (43.78)	32 (46.03)	1 (27.27)	
Infants characteristics				
Birth weight—*n*(%)				0.93
> =2500 g	564 (89.81)	350 (89.74)	13 (92.86)	
< 2500 g	64 (10.19)	40 (10.26)	1 (7.14)	
Survival state—*n*(%)				0.29
Died	11 (1.67)	7 (1.73)	1 (7.16)	
Survivors	647 (98.33)	398 (98.27)	13 (92.86)	
HIV Status—*n*(%)				0.011
Negative	633 (96.20)	394 (97.28)	11 (78.57)	
Positive	25 (3.80)	11 (2.72)	3 (21.43)	

### HIV status and associated-factors

Overall rate of HIV-vertical transmission was 3.59% (39/1086), and all infected children were enrolled for HIV care. According to feeding options, HIV transmission varied considerably: EBF (2.72%); ERF (3.80%); MF (21.43%), *p* = 0.011; without any significance (*p* = 0.34) between EBF and ERF (Table 1). Infants experiencing MF therefore had 5.3 and 7.4 folds higher risks of HIV MTCT compared to ERF and to EBF respectively.

As expected according to exposure to ARVs, HIV-vertical transmission rates were 1.7% (10/566) from ART-group, 1.9% (8/411) from AZT-group, and 19.2% (21/109) from ARV-naïve group, *p* < 0.0001.

**Fig. 2:**
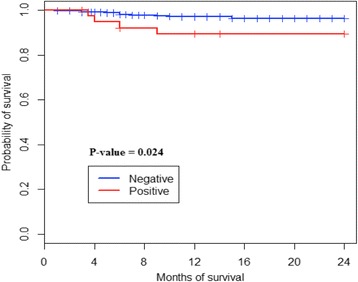
Kaplan-Meier cumulative probabilities curves of survival according to HIV status

According to HIV status, mortality rate was higher among HIV-infected (10.25%) vs. uninfected (2.29%) infants (*p* = 0.016), thus indicating about 4 folds increment for infected children. HIV negative children survived more than the positive ones (*p* = 0.024); the log-rank test also shows significant differences in the Kaplan Meier cumulative probability curves (Fig. 2). Similarly, the 24-month survival cumulative probability was varied considerably: 89.3% [79.9%-99.8%] vs. 96.4% [94.8%-97.9%] in infected vs. uninfected infants respectively, *p* = 0.024.

### Survival rates and associated-factors

Overall infant mortality in the study population was 2.58% (28), with varying trends according to feeding option: ERF (2.41%), EBF (2.45%), MF (13.33%), *p* = 0.032 (Table 2), indicating an increment of 5.4 and 5.5 folds in mortality for MF infants compared to EBF and ERF respectively. Similarly, the survival cumulative probability was 96% [94%-98%] in ERF, 96.4% [94.1%-98.8%] in EBF, and 86.67% [71.06%-100%] in MF, *p* = 0.04.

**Table 2:** Unadjusted and adjusted Hazard Ratio (HRs) of survival at 24 months in the HIV exposed infants of the Essos hospital center PMTCT program

	Unadjusted HR	*p*-value	Adjusted HR	*p*-value
Mode of feeding				
Exclusive breast feeding	1.35 [0.52-3.49]	0.54	1.16 [0.44-3.08]	0.76
Mixed feeding	0.31 [0.04-2.42]	0.26	0.44 [0.053-3.74]	0.45
Exclusive replacement feeding	Ref	Ref	Ref	Ref
HIV- Status				
Positive	0.31 [0.11-0.90]	0.032	0.30 [0.08-1.11]	0.071
Negative	Ref	Ref	Ref	Ref
Birth weight				
> =2500 g	0.70 [0.16-2.96]	0.63	0.93 [0.21-4.12]	0.92
< 2500 g	Ref	Ref	Ref	Ref
Global Schoenfeld residuals test				0.23

**Fig. 3:**
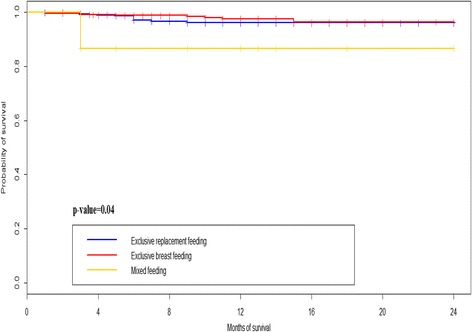
Kaplan-Meier cumulative probabilities curves of survival according to feeding option

Using the Log-rank test of survival (Fig. 3), a significant difference was observed in the Kaplan Meier curves according to feeding options (*p* = 0.04). Of note, the MF curve falls within the area under the curve and is distant from both the ERF and EBF curves. Specifically, the cumulative probability survival curve of MF compared those of EBF and EBF revealed *p*-values of 0.011 and 0.025 respectively. However, there was no significant difference of Kaplan Meier survival curves between EBF and ERF (*p* = 0.48). The 24-months survival cumulative probability was 96% [94%-98%] with ERF, 96.4% [94.1%-98.8%] with EBF, and 86.67% [71.06%-100%] with MF.

Schoenfeld residuals test revealed a proportional hazard assumption with similar effects of EBF and ERF on survivals (unajusted HR 1.30 [0.59- 2.85]; adjusted HR 1.25 [0.56-2.79]). Of relevance, children in the MF arm appeared to survive less than those on ERF (unadjusted HR 0.22 [0.05-0.96]). However, after adjustment for children HIV status, the effect of MF disappeared (Adjusted HR 0.29 [0.063-1.34]), insinuating that HIV-status of children is an intermediate independent factor between infant feeding option and survival.

## Discussion

In an attempt to ascertain the contributive effects of feeding options on HIV-1 vertical transmission and infant survival until 2 years of age, we demonstrated key outcomes of PMTCT programme performance after complete children follow-up throughout the PMTCT-cascade care in a high-priority country. In addition to the known impact of ARV [18, 20] within similar settings, feeding option appears as a contributive factor of infant clinical outcomes, thus paving the way for programmatic considerations [21, 22]. The present study therefore represents one of the few findings focusing on both vertical transmission and survival according to infant feeding options throughout the PMTCT-cascade care in a RLS. The findings of this study are likely to shift with the latest WHO recommendations which emphazise the practice of breastfeeding even after 12 months as recommended for the general population [21] and stresses the need not to interrupt breastfeeding in case of mixed feeding assuming maternal coverage of ARV.

The overall low rate of HIV-1 vertical transmission (3.59%) recorded in our study highlights the feasibility in achieving the national/global targets for virtual eMTCT (i.e. < 5%) [17, 18, 20], with better performance (i.e. < 2%) once PMTCT-interventions with ARV are effective [1–3]. In addition to previous knowledge, current findings underpin the significance of universal access to PMTCT option B+ [2, 6–9], with the potential of eMTCT globally [20].

Though practiced at a low rate (1.38%) in the study population, MF practice is still persistent in this RLS, indicated the need for advocacy towards continuous counseling of mothers for safe feeding options throughout the cascade of care, in order to mitigate the impact of unsafe feeding practice on PMTCT performance [21, 22]. In point of facts, beside ARV interventions, our observation places feeding option as a contributive factor for HIV vertical transmission, with potential impacts on the child overall health outcomes, including ERF in RLS [20–24].

In this urban RLS setting, higher rate of mortality was reported among HIV-positive children (compared to their negative counter parts) within the follow-up time of 24 months. This supports early pediatric ART initiation once diagnosed positive, to promote survival among infected children [25]. As the present evidence were generated in an urban setting, it is possible that mortality may be more burdened in rural settings whereby access to healthcare is more challenging in such SSA settings [26], thus suggesting wider or more representative assessments in varying geographical settings.

In the Cox model of multivariate analysis regarding feeding options and infant survival rates, MF appears as a significant contribution to poor survival events, thereby underlying the potential impact of MF on poor health outcomes of HIV-exposed infants [27, 28]. Thus, as safer feeding option, alongside ARVs, might serve as an additional weapon for both eMTCT while keeping the children alive, nutritional strategies to support maternal adherence to either ERF or EBF, using lessons of best feeding practitioners from successful PMTCT attendees, should be implemented for optimal outcomes [27–30].

Following the Schoenfeld residuals test of multivariate analysis, only HIV-infection was a predictor of infant mortality, with feeding option as a contributive factor. Thus, considering the reported strong association between feeding options and infant health outcomes (HIV MTCT and mortality events), it appears evident that MF contributes on both HIV MTCT and mortality among infants. Though the effect of MF is still being studied, this feeding option is assumed to impair the gut permeability, disturb the intestine microbiota, with exposure to many bacteria, microorganisms and antigens which may result in dysbiosis [31].

As EBF has better outcomes (though not significant) compared to ERF, the importance of EBF needs, in a context where access to potable water and other hygienic conditions are concerning, should be promoted extensively in RLS [32–36], for the overall health benefits of breast milk for the baby [36]. The practice of EBF over ERF has also been recommended by the WHO in their recent guidelines for RLS [20–22].

Overall strength of our findings relies on the association between survivals and the infant HIV status, with feeding option (i.e. MF) acting as an intermediary factor or vector of HIV transmission. Thus, considering generalizability within PMTCT high-priority countries, emphasis should be given to access to ART coupled to infant feeding counseling and support up to 2 years [21, 22]. The major strength of this study is the large sampling of our cohort coming from a SSA priority country, wherein fewer studies have reported on 2 years outcomes of HEI in routine settings. With over 70% retention in care, it would be relevant to investigate factors to improve retention also in HIV exposed uninfected infants, since they constitute a newly emerging epidemic group. Our analysis therefore demonstrates favorable outcomes at 24 months among HIV-exposed infants in both ERF and EBF arms, while those with MF showed the poorest outcomes.

A study limitation is the inability in ascertaining reasons justifying the reported cases of MF practices, which may be favoured by fear of rejection due to discrimination and stigma once HIV status is known. This stresses the role of health education, peer counselling, and male partner engagement as mitigating factors [37]. Also, lack of records for 6-months outcomes underscores the need to assess the impact of MF duration [38], as well as the infant nutritional status, on children health outcomes [39]. Furthermore, our study design did not give room for clinical follow-up, thus hindering the assessment of feeding practices at home, while missing data restricted the inclusion of some relevant factors in the survival analysis.

## Conclusions

Alongside PMTCT-interventions using ARVs, the practice of MF appears as a contributive factor to HIV-1 transmission to children. Furthermore, beside the infant HIV status, the practice of MF also seems to contribute on mortality events among children. Specifically, the core role of HIV status for survival, added to the mitigating effect of feeding option, makes mandatory the infant counseling during the first 6 months of life. Therefore, in the era of option B+, continuous counseling on best feeding practices throughout the PMTCT-cascade needs to be strengthened in RLS. Such policy would help in further eliminating new MTCT while improving the life expectancy of children within PMTCT high-priority countries.

## Abbreviations

ANC: Antenatal care
ART: Antiretroviral therapy
ARV: Antiretroviral
AZT: Zidovudine
EBF: Exclusive breastfeeding
EHC: Essos Hospital Centre
eMTCT: Elimination of mother-to-child transmission of HIV
ERF: Exclusive replacement feeding
IFC: Infant feeding counseling
MF: Mixed feeding
PMTCT: Prevention of mother-to-child transmission of HIV
RLS: Resource-limited settings
SSA: Sub-Saharan Africa
